# Experimental and Numerical Investigations on the Seismic Performance of High-Strength Exterior Beam-Column Joints with Steel Fibers

**DOI:** 10.3390/ma17164066

**Published:** 2024-08-16

**Authors:** Bingliu Wu, Xingjian Liu, Junyu Jia, Deming Fang, Jianwen Shao, Wei Kong

**Affiliations:** 1 The Architectural Design & Research Institute of Zhejiang University Co., Ltd., Hangzhou 310058, China; tmgcwbl2005@163.com; 2 Center for Balance Architecture, Zhejiang University, Hangzhou 310058, China; 3 College of Civil Engineering, Zhejiang University of Technology, Hangzhou 310023, China; 17326082478@163.com (X.L.); fdeming@163.com (D.F.); 4 Zhejiang Construction Engineering Quality Inspection Station Co., Ltd., Hangzhou 310012, China; 5 Key Laboratory of Performance Evolution and Control for Engineering Structures of Ministry of Education, Tongji University, 1239 Siping Rd., Shanghai 200092, China; junyu_jia@126.com; 6 Department of Digital Construction, Shanghai Urban Construction Vocational College, Shanghai 200438, China; 7 Zhejiang Jinggong Steel Building Group Co., Ltd., Shaoxing 312030, China; 8 College of Urban Construction, Zhejiang ShuRen University, Hangzhou 310015, China; 9 China Railway Construction East China Co., Ltd., Kunshan 213500, China; csjstructure2021@126.com

**Keywords:** beam-column joint, steel fibers, high-strength concrete, seismic performance, hysteresis response, ductility

## Abstract

Steel fiber reinforced high-strength concrete (SFRHSC) is a composite material composed of cement, coarse aggregate, and randomly distributed short steel fibers. The excellent tensile strength of steel fiber can significantly improve the crack resistance and ductility of high-strength concrete (HSC). In this study, experimental and numerical investigations were performed to study the cyclic behavior of the HSC beam-column joint. Three SFRHSC and one HSC beam-column joint were prepared and tested under cyclic load. Two different volume ratios of steel fibers and three stirrups ratios in the joint core area were experimentally studied. After verification of the experimental results, numerical simulations were further carried out to investigate the influence of steel fibers volume ratio and stirrups ratio in the joint core area on the seismic performance. Evaluation of the hysteretic response, ductility, energy dissipation, stiffness, and strength degradation were the main aims of this study. Results indicate that the optimal volume fraction of steel fibers is 1.5%, and the optimal stirrups ratio in the joint core area is 0.9% in terms of the enhancement of the seismic performance of the SFRHSC beam-column joint.

## 1. Introduction

The beam-column joints are the bearing members in the frame structure. Under seismic load, ensuring the reliability of structures is of top priority, where the beam-column joints are required to have excellent seismic performance [1,2]. As such, the design requirements of “strong joint members” have been proposed [3]. It requires the core area of beam-column joints to embed a considerable amount of stirrups. However, this inevitably leads to problems such as the difficulty of bending steel bars in the core area, the difficulty of vibration and compaction of concrete, and the increase in economic costs. In this regard, research has been conducted on the methods to improve the seismic performance of concrete, such as ductility and energy dissipation capacity, while avoiding the issues mentioned above [4,5,6]. The incorporation of different types of fibers into high-strength concrete (HSC) is one of the most effective methods [7,8,9].

Fiber effectively inhibits the generation and development of concrete shrinkage cracks [10,11], among which steel fiber is widely used due to its effect on improving the mechanical properties and deformation resistance of concrete [12]. Studies have shown that the addition of steel fiber improves the overall working performance of concrete structures and optimizes the ductility and energy dissipation capacity [13,14]. With the development of infrastructure, HSC with large span and high strength has developed rapidly [15], but HSC is also a brittle material with low tensile strength and poor ductility. Steel fiber reinforced high-strength concrete (SFRHSC) is a composite material composed of cement, coarse aggregate, and randomly distributed short steel fiber. The excellent tensile strength of steel fiber improves the cracking resistance and ductility of HSC and optimizes its seismic performance [7]. SFRHSC possesses high flexural and shear strength, in addition to superior resistance to compression. It can be either precast or poured in situ. Therefore, incorporating steel fiber into concrete structures and its synergistic effect to resist seismic load become a hot topic.

Factors affecting the seismic performance of beam-column joints of structures include the strength of the concrete matrix [16], stirrups ratio in the joint core area [17], axial compression ratio [18], bonding and anchoring performance of beam-end longitudinal reinforcement, geometric dimensions [19]. For instance, the ductility of an HSC column-normal strength concrete slab structure is significantly affected by the dimension of the column’s section [19]. With the increase of the steel fibers volume ratio, the seismic performance indexes (i.e., ductility, energy dissipation capacity, bearing capacity, and shear deformation resistance) are obviously improved [20,21]. The seismic performance of the specimens with steel fibers in the beam-end of the beam-column joint is better than that of the specimens without steel fibers in the beam-end region. Generally, the factors affecting the seismic performance of HSFRC beam-to-column joints have been defined. Nevertheless, the optimal volume content of steel fibers and the stirrups ratio in the joint core area have not obtained a relatively consistent conclusion. Additionally, whether steel fibers can replace the stirrups in the joint core area and the percentage of replacement are still in question.

In this study, three SFRHSC and one HSC beam-column joint were designed and fabricated. The main variables were the volume ratio of steel fibers and the stirrups ratio in the joint core area. A cyclic loading test was carried out. The failure modes, shear deformation in the joint core area, deformation in the plastic hinge area of the beam-end, reinforcement strain, hysteresis response, skeleton curve, ductility, energy dissipation capacity, stiffness, and strength degradation were analyzed. Based on verification by experimental results, numerical simulations were further performed. The aim of this study is to reduce the number of stirrups in the core area of beam-column joints by incorporating steel fibers to improve seismic performance. Suggestions for the design and application of SFRHSC in frame structures can thus be provided.

## 2. Experimental Program

### 2.1. Specimen Preparation

Four high-strength concrete (HSC) beam-column joint specimens were fabricated to investigate the seismic performance of high-strength exterior beam-column joints with steel fibers, including three HSC joints containing steel fibers (SFRHSC) and one HSC beam-column joints without steel fibers.

**Figure 1:**
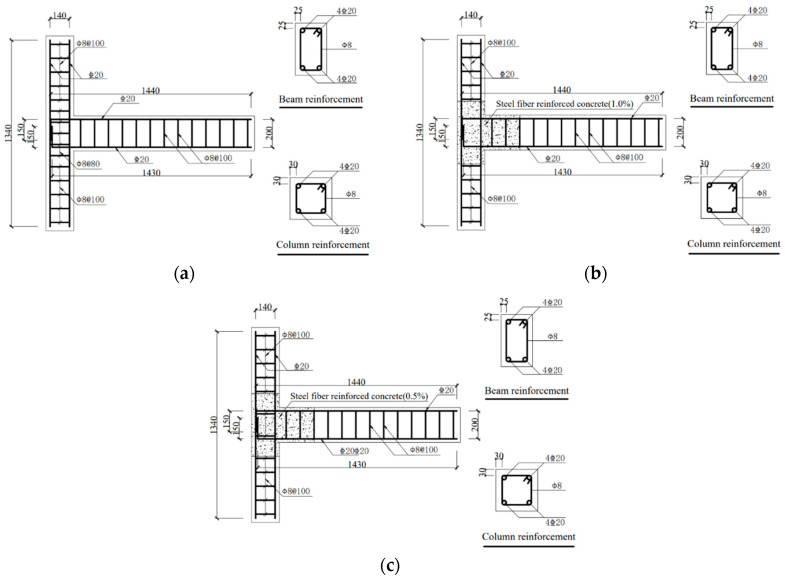
Dimensions of beam-column joint specimens for (**a**) KJ1-1, KJ1-2, (**b**) KJ2-1, (**c**) KJ2-2 (unit: mm). The shadow part in the figure represents steel fiber concrete, and the blank part in the figure represents concrete without steel fibers.

Beam-column joint specimens were designed according to Chinese standards [22,23,24]. The details of joint specimens are presented in Table 1. The volume of steel fibers, around 1.0%, enhances the mechanical properties of concrete while not dramatically decreasing its workability [25]. Therefore, the volume ratio of 1.0% steel fibers in the joint core area is used in the experiments. Figure 1 illustrates the dimensions of each joint specimen. Specimens KJ1-1 and KJ1-2 were tested to investigate the influence of different steel fiber ratios. The effect of the stirrups ratio in the joint core area was studied through KJ1-2, KJ2-1, and KJ2-2.

The beam-column joint specimens were cast horizontally. In the joint core area and the plastic hinge area of the beam-end, steel fibers were incorporated into concrete, as depicted in the shadow region in Figure 1b,c. A baffle plate was used to separate HSC and SFRHSC. After the concrete was filled within the mold, the baffle plate was pulled out.

### 2.2. Properties of Materials

The tensile strength of hooked-end steel fibers used in this study is 1291 MPa, with a length of 35.01 mm and a diameter of 0.55 mm. Table 2 lists the mixture proportion of concrete with two different proportions of steel fibers (i.e., 0.0% and 1.0%). Concrete specimens were cast to obtain their mechanical properties, as summarized in Table 3. Three 150 mm × 150 mm × 150 mm cubes were cast for measuring the cubic compressive strength. Three 150 mm × 150 mm × 150 mm cubes for splitting tensile strength test. Three 150 mm × 150 mm × 300 mm prismatic blocks were prepared to obtain the axial compressive strength. Three 150 mm × 150 mm × 300 mm prismatic blocks for elastic modulus measurement. Prior to the loading test of the beam-column joint, the mechanical properties of concrete specimens were tested after 28 days of curation. The values listed in Table 3 are the average value of three concretes in each mechanical test. The plain steel with a diameter of 8 mm was used as the stirrups. The ribbed steel with a diameter of 20 mm was employed as the main rebar. The mechanical properties of steel reinforcement are listed in Table 4.

Due to the large size of the joint specimen and its difficulty in moving, the beam-column joint specimen and concrete test blocks were treated by outdoor wrapped watering. The beam-column joint specimens and concrete test blocks were wrapped with burlap and watered three times a day in the morning, noon, and evening. After pouring the water, the specimens and test blocks were covered with plastic tape to reduce water evaporation. The temperature during curation ranged between 5 and 18 °C.

### 2.3. Test Setup and Loading System

**Figure 2:**
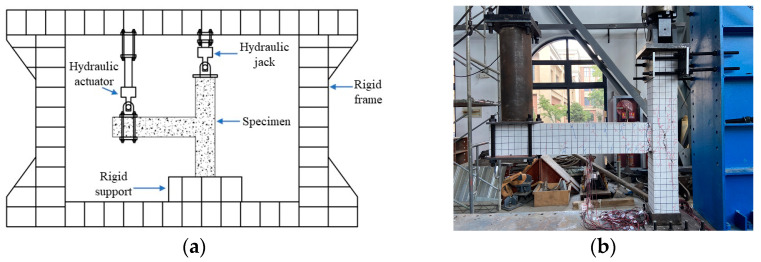
(**a**) Schematic illustration of loading system and (**b**) Joint specimen KJ2-1.

The loading device used in this study is a pseudo-static electro-hydraulic servo loading system, including a 100 t single-ejector lever servo actuator and a 60 t single-ejector lever servo actuator with a stroke of 600 mm. The test device consists of a reaction frame, a rigid support, and a loading connection device for the electro-hydraulic servo loading system, as schematically shown in Figure 2.

**Figure 3:**
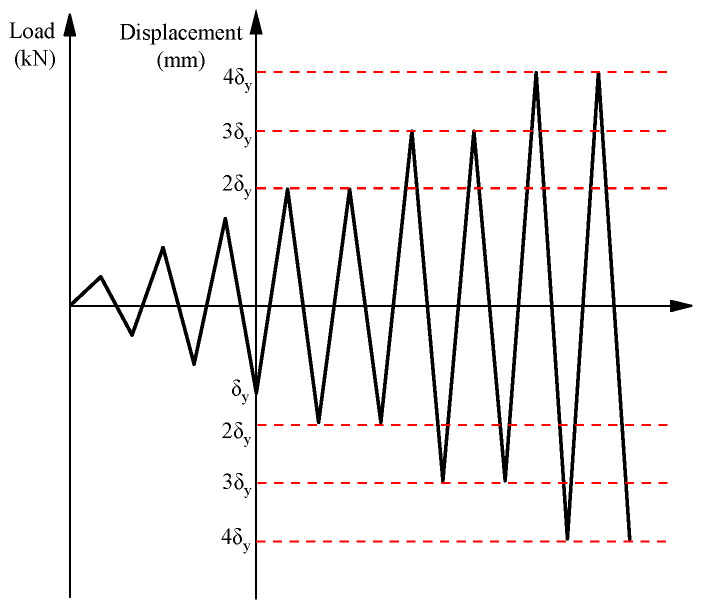
History of loading.

To study the influence of seismic load on beam-column joints, constant axial pressure was applied to the top of the beam-column joints; cyclic load was applied to the beam ends. Notedly, the structural performance of beam-column joints can also be affected by the axial force [26]. Increasing the axial load possibly enhances the joint shear strength thanks to the increase in the depth of the compressive strut. Given that the effect of axial force on the seismic performance of joints is not the scope of this work, the level of axial load was kept constant in the present study. The internal force of the loading joints was transmitted to the plastic hinge area of the beam ends and the core area of the column through the beam ends, which conformed to the transmission path of seismic loads. According to Chinese standards (JGJ/T101-2015) [27], the loading method adopted load-displacement joint control. Before the bearing capacity of the beam-column joint reached the yield load, the test adopted load-controlled loading. Afterward, the test adopted displacement-controlled loading. Figure 3 demonstrates the loading history. Prior to the loading test, a numerical simulation was performed to obtain the initial yield load of the beam-column joints. A 3-level load control was set for the loading test. During the loading process, the strain data of the main rebar at the beam-column joint was observed simultaneously. When the data of the steel strain gauge reached the yield strain, the load value was recorded as the yield load *P*_y_, and the displacement value of the beam-end was the yield displacement *δ*_y_. The loading of the beam-column joint was then changed from a load-controlled to a displacement-controlled process. The loading test stopped when the joint specimens were damaged, or the specimens appeared to have huge deformation or cracks and were unable to bear the load, or the bearing capacity of the specimens decreased to less than 85% of the peak test load.

### 2.4. Instrumentation and Crack Mapping

**Figure 4:**
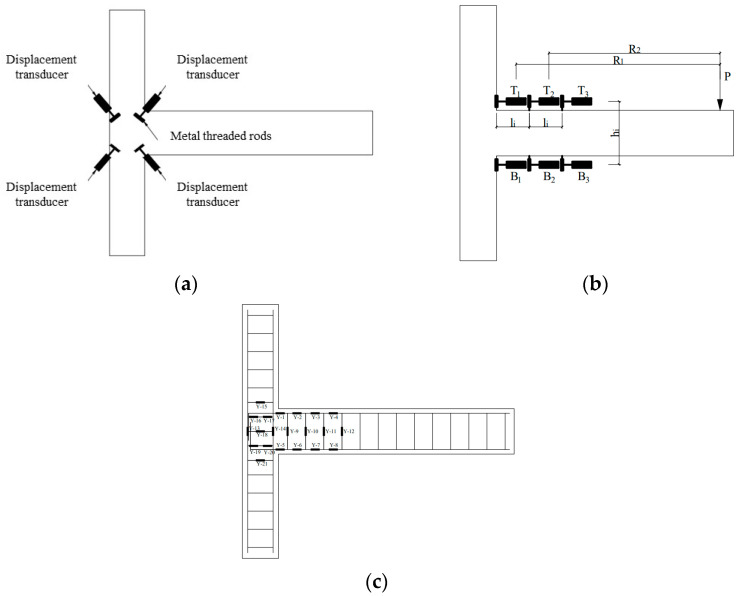
Arrangement of (**a**,**b**) displacement transducers on the joint, and (**c**) strain gauge of steel. T_1_, T_2_, T_3_, B_1_, B_2_, and B_3_ are the displacement values of the upper and lower sides of the plastic hinge area of the beam end. h_1_ and h_2_ are the height difference of the displacement transducers on the upper and lower sides of the beam end, which is 270 mm; l_1_ and l_2_ are the displacement transducers layout intervals, l_1_ = l_2_ = 200 mm.

A total of ten displacement transducers were installed to measure the displacement of different positions of the joint. Four displacement transducers were arranged along the diagonal direction of the joint core area. As illustrated in Figure 4a, transducers in the diagonal direction were aligned on the same straight line, while those in the other direction were arranged perpendicular to each other. To provide attachment for the transducers, holes were drilled in joint specimens, and metal threaded rods were inserted. By measuring the real-time data from the displacement transducers, the shear deformation in the core area was obtained. Three displacement transducers were arranged on the upper and lower sides of the plastic hinge areas of the beam-column joints, respectively. The arrangement of displacement transducers is demonstrated in Figure 4b, with 0 mm, 200 mm, and 400 mm away from the column edge.

To study the development trend of the stress-strain of reinforcements as the load changed, the strain gauges were polished and arranged on the stirrup and longitudinal reinforcement in the core area and the plastic hinge area of the beam-end (Figure 4c).

Prior to the loading test, the surface of joint specimens was painted white with latex paint, and a 50 mm × 50 mm grid was drawn, as shown in Figure 2b. At the end of each loading stage of the joint specimens, the shape and development trend of cracks were drawn on the specimens’ surface, and the loading level when cracks appeared was recorded. After the loading test, the maximum width of each crack on the surface of the beam-column joint specimens was measured using a crack width gauge to analyze the crack development and failure characteristics of the specimens.

## 3. Experimental Results

### 3.1. Crack Patterns and Failure Mode

**Figure 5:**
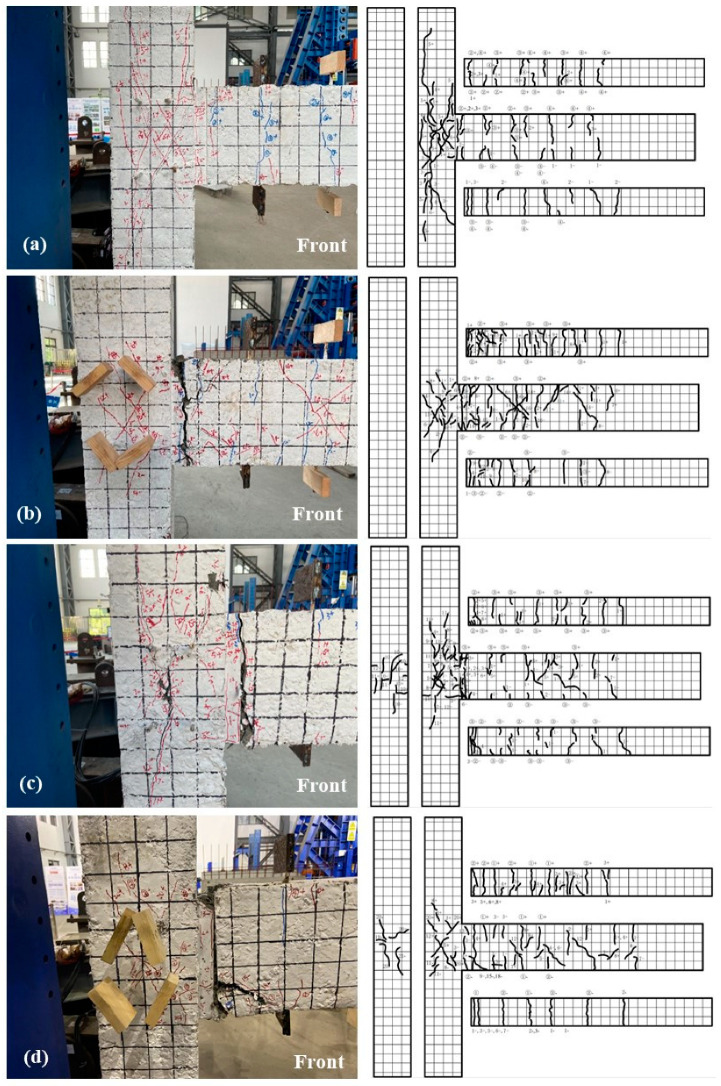
The front crack pattern of the joint specimen (**a**) KJ1-1, (**b**) KJ1-2, (**c**) KJ2-1, and (**d**) KJ2-2.

Figure 5 shows the crack patterns of each beam-column joint specimen. By analyzing the crack development trend and final failure of joint specimens during the loading process, it was concluded that the predominant failure modes of beam-column joint specimens are core shear failure and beam-end flexural failure.

Specimens KJ1-1 and KJ2-1 exhibited shear failures within the core area with the following features: A series of oblique and parallel cracks emerged near the middle section of the core area. Simultaneously, the majority of the stirrups within this region yielded while localized spalling occurred in the concrete protective layer. Meanwhile, cracks at the beam end remained relatively small but steadily propagated as they concentrated primarily within the joint’s core area (Figure 5a,c). On the contrary, specimens KJ1-2 and KJ2-2 exhibit beam-end flexural failures (Figure 5b,d). Longitudinal steel bars at these specimens’ beam ends yielded. Annular cracks significantly developed, leading to concrete spalling and exposure of reinforcing steel. As vertical cracks expanded further, a series of oblique cracks appeared until they eventually connected the upper and lower ends to form plastic hinges. However, cracks within these joints’ core areas progressed slowly, with only minor cracks observed upon destruction.

Table 5 lists the crack parameters of specimens. During the early stage of loading, the addition of steel fibers delayed the onset of cracking and reduced the crack width. For specimens subjected to beam-end flexural failure (i.e., KJ1-2 and KJ2-2), the maximum crack width in the core area remained at a relatively small value.

### 3.2. Deformation and Strain Analysis

#### 3.2.1. Shear Deformation in the Core Area

**Figure 6:**
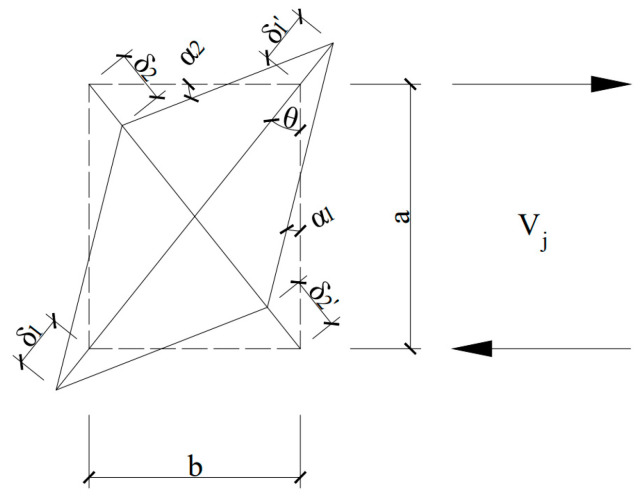
Schematic diagram of calculating shear angle *γ*.

The shear deformation caused by concrete cracking in the joint core area is the main factor affecting the seismic performance of the beam-column joint. The shear deformation in the core area was measured by the change in the diagonal length of the core area (Figure 4a). As schematically illustrated in Figure 6, the rectangle represents the core area of the bear-column joint within the range of the main reinforcement. Under the action of horizontal shear Vj, one diagonal direction extends (*δ*_1_′ + *δ*_1_), and the other diagonal direction shortens (*δ*_2_′ + *δ*_2_), resulting in angles (*α*_1_, *α*_2_, and *θ*) after deformation. Joint shear deformation can be characterized by the shear angle *γ*, which was calculated through Equations (1)–(4).

(1) $$\bar{X}=\frac{\left(\delta_{1}^{\prime }+\delta_{1}\right)+\left(\delta_{2}^{\prime }+\delta_{2}\right)}{2}$$

(2) $$\sin\theta =\frac{b}{\sqrt{a^{2}+b^{2}}}\;\cos\theta =\frac{a}{\sqrt{a^{2}+b^{2}}}$$

(3) $$\alpha_{1}=\frac{\bar{X}\sin\theta }{a}\;\alpha_{2}=\frac{\bar{X}\cos\theta }{b}$$

(4) $$\gamma =\alpha_{1}+\alpha_{2}=\frac{\bar{X}\sin\theta }{a}+\frac{\bar{X}\cos\theta }{b}=\frac{\sqrt{a^{2}+b^{2}}}{ab}\times \bar{X}$$

where $\bar{X}$ is the average change along the diagonal direction of the core area; *δ*_1_, *δ*_1_′, *δ*_2_, and *δ*_2_′ are the four displacement meter values on the diagonal of the core region respectively; *α*_1_ and *α*_2_ are the angles after the diagonal deformation of the core region; *a* and *b* are the cross-section dimensions of the core area, *a* = 250 mm, *b* = 200 mm; *γ* is the shear angle generated after the shear deformation of the core region.

**Figure 7:**
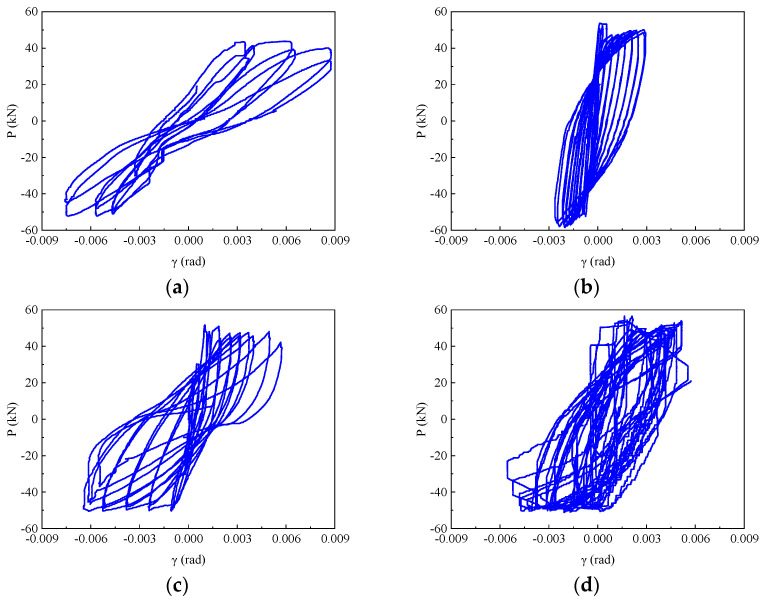
*P*-*γ* diagram of beam-column joint (**a**) KJ1-1, (**b**) KJ1-2, (**c**) KJ2-1, and (**d**) KJ2-2.

The results of the load-shear angle (*P*-*γ*) diagram of the beam-column joint are demonstrated in Figure 7. At the initial stage of loading, the *P*-*γ* diagram of all beam-column joint specimens showed a similar trend, which was a linear relationship. Nevertheless, with the increase in loading level, the shear angle development of beam-column joints with different failure modes behaved dissimilarly. For the specimens KJ1-1 and KJ2-1 with shear failure in the core area, large shear deformation was demonstrated in the shear angle development (see Figure 7a,c). The *P*-*γ* curve was pinched and showed an inverted S-shape. For the specimens KJ1-2 and KJ2-2, which suffered a beam-end flexural failure, the shear angle developed statically, with small variation amplitude and small shear deformation. In the later period of loading, the *P*-*γ* curve remained full and developed in a linear trend.

**Figure 8:**
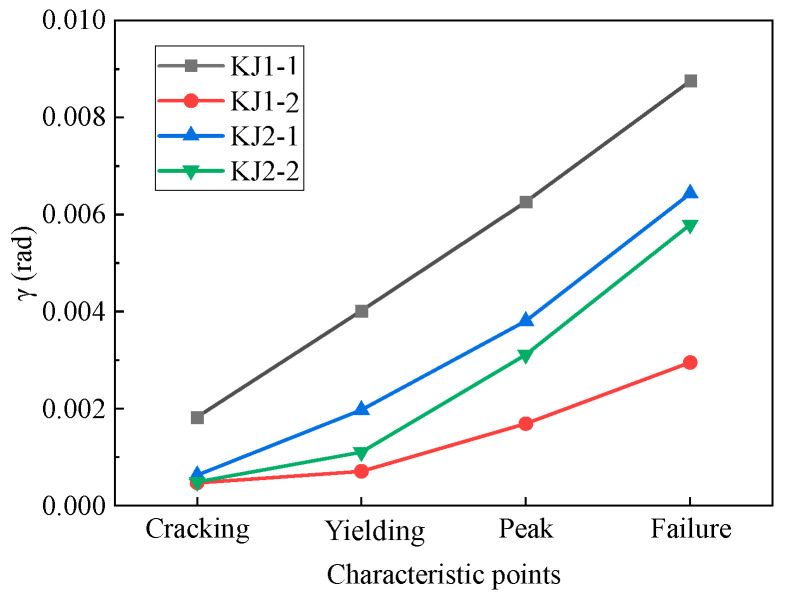
The shear angle of characteristic points of beam-column joints.

Then, shear angles at characteristic points (i.e., the points of cracking, yielding, peak value, and failure) of each beam-column joint during loading were extracted from Figure 7. It can be seen from Figure 8 that the shear angle of KJ1-2 at the failure stage was reduced by 66.7% compared with that of KJ1-1. The inclusion of steel fibers in the joint core area significantly constrains the shear deformation of the core area. The steel fibers can effectively inhibit the cracking of concrete (i.e., delay the cracking time and reduce the crack parameters like the number and length of cracks) [28,29], as can be seen in Table 5. The ultra-high tensile strength of steel fibers ensures the working performance of concrete after cracking and weakens the local damage.

By replacing the stirrups in a core area with steel fibers, the shear angle of specimen KJ2-1 ranged from −0.00643 to 0.00643 (Figure 7c), and the shear angle of failure characteristic points was reduced by 26.5% (Figure 8). This is due to the fact that the dense and disordered addition of steel fibers into the concrete effectively replaces or even enhances the anti-shear function of the stirrup in the core area. In the case of partial replacement of stirrups with steel fibers, the shear angle of specimen KJ2-2 ranged from −0.00579 to 0.00579. The shear angle of failure characteristic points was reduced by 33.8% compared to KJ1-1. This indicates that partial replacement can be more effective than complete replacement in terms of shear deformation in the joint core area.

#### 3.2.2. Deformation of Plastic Hinge Area at Beam-End

Under seismic load, the beam-column joint is subjected to cyclic load, and the beam-end near the column core area gradually forms a plastic hinge after yielding. The rotation of the plastic hinge area of the beam-end is the main factor affecting the deformation of the beam-column joint.

The average curvature *ϕ* was used to represent the rotation of the plastic hinge area of the joints, which was calculated through Equations (5) and (6).

(5) $$\phi_{1}=\frac{\left|T_{2}-T_{1}\right|+\left|B_{2}-B_{1}\right|}{h_{1}l_{1}}$$

(6) $$\phi_{2}=\frac{\left|T_{3}-T_{2}\right|+\left|B_{3}-B_{2}\right|}{h_{2}l_{2}}$$

where *ϕ*_1_ and *ϕ*_2_ are the average curvature of the plastic hinge area of the beam-end in the range of 0~200 mm and 200~400 mm from the column edge, respectively; *T*_1_, *T*_2_, *T*_3_, *B*_1_, *B*_2_, and *B*_3_ are respectively the displacement meter values of the upper and lower sides of the plastic hinge area of the beam end; *h*_1_ and *h*_2_ are the height difference of the displacement meter on the upper and lower sides of the beam end, which is 270 mm; *l*_1_ and *l*_2_ are the displacement meter layout intervals, *l*_1_ = *l*_2_ = 200 mm (see Figure 4b).

**Figure 9:**
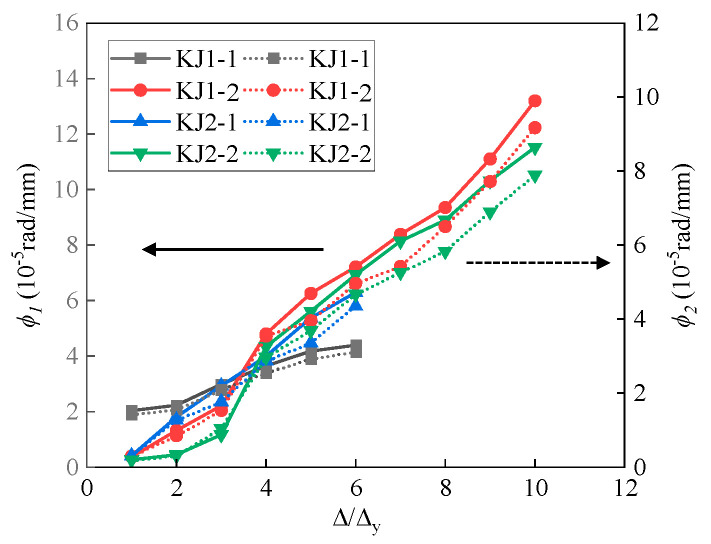
Average curvature of plastic hinge area of the beam-column joint. The solid line represents *ϕ*_1_, while the dashed line represents *ϕ*_2_.

The results of the mean curvature *ϕ*_1_ and *ϕ*_2_ in the plastic hinge area are shown in Figure 9. The Δ_y_ denoted the displacement when the control mode of loading changed from load-controlled to displacement-controlled procedure. The value of Δ/Δ_y_ represented the loading level, which was the ratio of displacement amplitude to yield displacement. The maximum *ϕ*_1_ and *ϕ*_2_ of specimen KJ1-1 were 4.40 × 10^−5^ rad/mm and 3.11 × 10^−5^ rad/mm, respectively. The addition of steel fibers in the joint core area significantly increased the curvature, as shown in the results of KJ1-2 and KJ2-2. The mean curvature of *ϕ*_1_ and *ϕ*_2_ for specimens KJ1-2 and KJ2-2 were 10.34 × 10^−5^ rad/mm and 7.13 × 10^−5^ rad/mm, which were 135.0% and 129.2% higher than that of the specimen KJ1-1. It indicates that the addition of steel fibers dramatically enhances the rotation ability of the plastic hinge area at the beam end of the joint. For specimens KJ2-2 and KJ2-1, whose stirrups in core area were partly or completely replaced by steel fibers, the maximum *ϕ*_1_ and *ϕ*_2_ of KJ2-2 were 11.52 × 10^−5^ rad/mm and 7.89 × 10^−5^ rad/mm, while the maximum *ϕ*_1_ and *ϕ*_2_ of KJ2-1 were 6.29 × 10^−5^ rad/mm and 4.35 × 10^−5^ rad/mm. It indicates that partial replacement of stirrups in the joint core area exhibits superior performance compared to complete replacement. Nevertheless, completely replacing the stirrups with steel fibers still yields better rotation ability than the HSC joint.

Additionally, the average curvature of specimen KJ1-1 developed slowly with the increase in loading level, and the slope of the curve tended to be flat in the later period of the loading test. Contrarily, the slope of the curve for the other three specimens with steel fibers tended to increase gradually with the increase in loading level. After the loading level of 3Δ_y_, the average curvature of the plastic hinge area of joints KJ1-2, KJ2-1, and KJ2-2 exceeded specimen KJ1-1. The state at 3Δ_y_ corresponded to the cracking load stage, indicating that the addition of steel fiber not only enhanced the rotation ability of the plastic hinge area but also maintained the working performance after concrete cracking.

#### 3.2.3. Stirrups Strain in the Core Area

**Figure 10:**
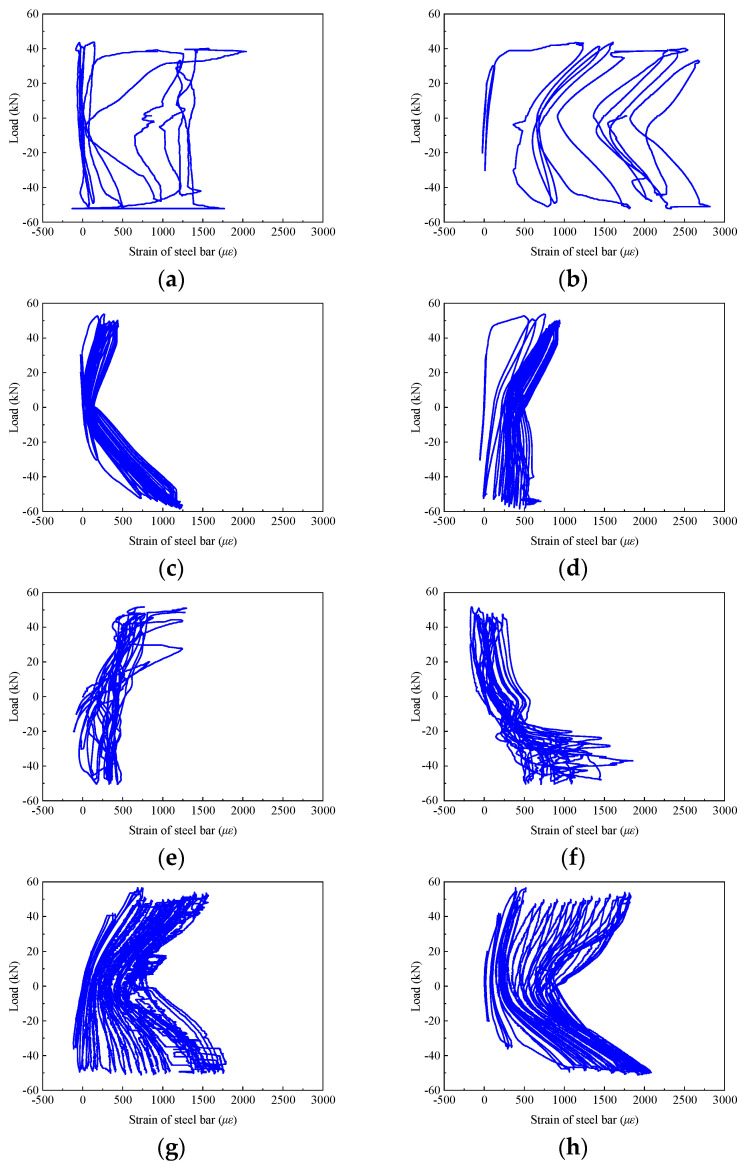
Stirrup strain in the core area for the specimen (**a**) KJ1-1/Y-16, (**b**) KJ1-1/Y-20, (**c**) KJ1-2/Y-16, (**d**) KJ1-2/Y-20, (**e**) KJ2-1/Y-13, (**f**) KJ2-1/Y-14, (**g**) KJ2-2/Y-16, (**h**) KJ2-2/Y-20. The position of the strain gauge is referred to in Figure 4c.

Figure 10 shows the strain curve of the stirrup in the core area of each beam-column joint as the load changed, which was denoted by Y-16 and Y20 with the positions shown in Figure 4c. The core area of specimen KJ2-1 was not equipped with stirrups. As such, the longitudinal reinforcement strains Y-13 and Y-14 were demonstrated. As can be seen, the stirrup strain in the core area of KJ1-1 demonstrated the fastest increase with increasing the loading level. The stirrup strain in the core area of KJ1-1 reached the yield strain of 2100 *με* before the end of the test and eventually reached 2750 *με*. With the addition of steel fibers, the strain of specimen KJ1-2 developed slowly, and the load-strain curve of the reinforcement showed a pinching trend. At the end of the loading test, the maximum strain of the stirrup in KJ1-2 was 1250 *με*, which had not yet reached the yield strain of the stirrup. It is suggested that the stirrups ratio in SFRHSC should not be too large. Further investigations regarding the effect of stirrups ratio are discussed in the following parametric analysis section.

Overall, the steel fibers directly bear the shear force of the stirrup in part of the core area, restrain the shear deformation of the concrete, maintain the integrity of the joint core area, and slow down the development of stirrup strain.

### 3.3. Hysteresis Response and Skeleton Curves

**Figure 11:**
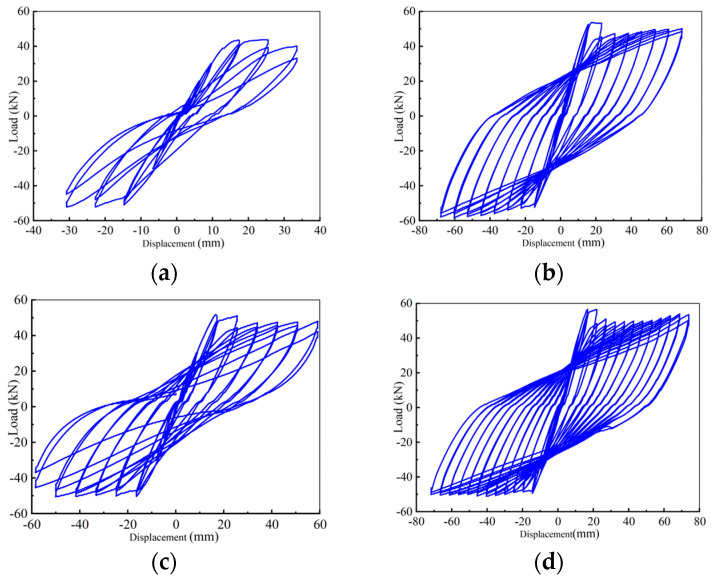
Hysteresis curves of beam-column joint specimen (**a**) KJ1-1, (**b**) KJ1-2, (**c**) KJ2-1, and (**d**) KJ2-2.

The shape of the hysteresis curve can comprehensively reflect various seismic performance indexes such as ductility, energy dissipation capacity, bearing capacity degradation, and stiffness degradation. Figure 11 demonstrates the hysteretic curves of beam-column joint specimens under seismic load with the following characteristics: (1) In the initial stage of loading, the hysteretic curves were relatively pinched. The load-displacement relationship was linear, indicating that the specimens were in the elastic stage; (2) With the increase of load, the steel bar at the end of the beam yielded, the envelope area of the hysteresis curve increased, and the hysteresis ring became fuller. At this moment, the specimen began to crack. The steel fibers in concrete played a bridging role, which increased the energy dissipation capacity and the bearing capacity of the specimen. The specimen turned into the strengthening stage. (3) After the load reached the peak load, the bearing capacity of the specimen reached the limit, and the bearing capacity of the hysteresis curve began to decline. At this moment, the joint was seriously damaged. The stiffness degradation of the joint gradually decreased, and the area of the hysteresis ring reduced. The specimen was in the failure stage.

The specimen KJ1-1 demonstrated a Z-shaped hysteresis curve (Figure 11a). It indicates that the energy dissipation capacity of KJ1-1 is poor. The load decreased rapidly after reaching the peak load, and the stiffness of the specimen decreased rapidly in the failure stage. Eventually, the ultimate bearing capacity and ultimate displacement were minimal. The structure presented the worst displacement ductility performance. With the addition of steel fibers, the hysteresis curve of specimen KJ1-2 was full, indicating a stronger energy dissipation capacity than KJ1-1 (Figure 11b). The load decreased slightly after the peak load. Although the concrete on the surface of the specimen was damaged and spalling, the embedded steel fibers tightly pulled the concrete on both sides of the crack, exerting the “bridge function” of the steel fiber, as reflected in the hysteresis curve. As such, the bearing capacity of the specimen still rose until the failure stage.

When replacing all stirrups in the core area with steel fibers, as can be seen in Figure 11c, the hysteresis curve of KJ2-1 was full and pinching compared with KJ1-1, demonstrating an inverted S-shape of the hysteresis ring. The load began to decline slowly after reaching the peak load, and the stiffness slowly declined from the failure stage. The descending speed was slower than KJ1-1. The ultimate bearing capacity, ultimate displacement, and structural displacement ductility were improved compared with KJ1-1. When partially replacing stirrups in the core area with steel fibers, as shown in Figure 11d, the hysteresis curve of KJ2-2 was full, and the hysteresis ring was arcuated, indicating a strong energy dissipation capacity. The decline of bearing capacity and stiffness showed the same trend as that of KJ1-2.

By comparing the hysteretic curves of specimens KJ1-1 and KJ1-2, it can be concluded that the hysteretic curves of SFRHSC beam-column joints are fuller, and the seismic performance is improved. By comparing the hysteretic curves of specimens KJ1-1, KJ2-1, and KJ2-2, it can be concluded that the hysteretic curve does not show a pinch phenomenon when the stirrups reinforcement in the core area is replaced by steel fiber. Compared with specimen KJ1-1 without steel fiber, the overall performance is better, the shape of the hysteretic ring is fuller, and the shear resistance is stronger. The bearing capacity, stiffness, and structural displacement ductility are improved, which indicates that steel fiber can replace the stirrup in the core area of the beam-column joint.

**Figure 12:**
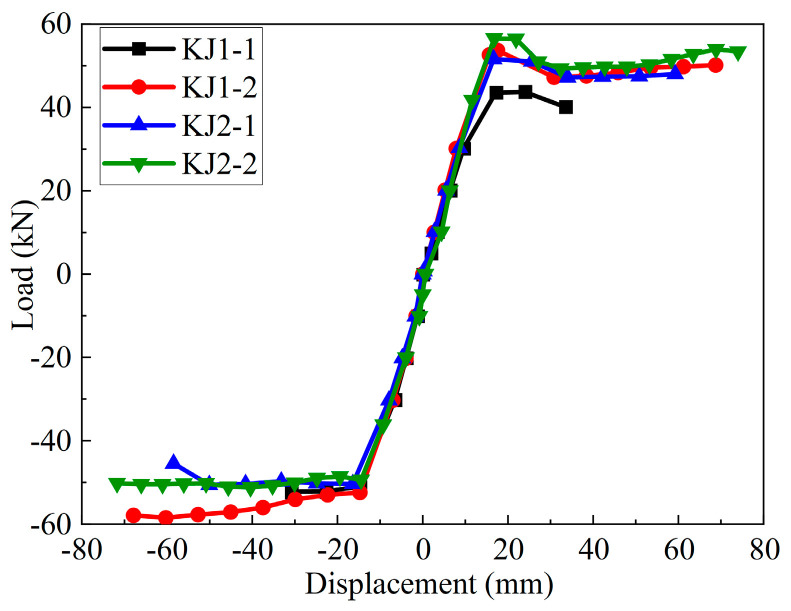
Skeleton curves of beam-column joints.

The skeleton curve is a load-displacement curve formed when each stage of the hysteretic curve is connected at the peak point of the first cyclic load. It reflects the bearing capacity and ductility performance of the component. As shown in Figure 12, with the addition of steel fibers, the slope of the skeleton curve increased during the elastic loading stage. The initial stiffness of beam-column joint specimens was improved. During the loading and strengthening stage, the peak load and peak displacement of specimens KJ1-2, KJ2-1, and KJ2-2 were larger than that of specimen KJ1-1. In the loading failure stage, specimen KJ1-1 rapidly failed after the bearing capacity decreased, while specimens KJ1-2, KJ2-1, and KJ2-2 maintained certain working performance after the bearing capacity decreased. The ultimate load and ultimate displacement of KJ1-2, KJ2-1, and KJ2-2 were greater than that of specimen KJ1-1.

Table 6 lists the characteristic values analyzed through skeleton curves. After the addition of steel fibers, the cracking load (*P*_cr_) of KJ1-2 was significantly increased by 50%, the yield load (*P*_y_), peak load (*P*_max_), and ultimate load (*P*_u_) were increased by 24.2%, 16.9%, and 32.5%, respectively. By comparing the joints KJ1-1, KJ2-1, and KJ2-2, it can be found that when the stirrup in the core area is partially or completely replaced by steel fibers, the values of *P*_cr_, *P*_y_, *P*_max_, and *P*_u_ were all increased. It indicates that the steel fibers can replace the stirrup in the joint core area, demonstrating a better bearing capacity of the joint.

### 3.4. Ductility

Based on the energy equivalent method [30,31], the yield displacement, ultimate displacement, and displacement ductility coefficients of the four beam-column joints were obtained. The displacement ductility coefficient *μ* was calculated through Equation (7). As listed in Table 6, the displacement ductility coefficient *μ* was significantly improved by the addition of steel fibers. Compared with specimens KJ1-1, the average value of displacement ductility coefficients for forward and reverse loading of specimens KJ1-2, KJ2-1, and KJ2-2 were increased by 38.0%, 24.8%, and 32.5%, respectively. Noticeably, when the stirrups in the core area were partially or completely replaced by steel fibers, the displacement ductility coefficient significantly increased. Steel fibers can replace the stirrups in the core area with better ductility.

(7) $$\mu ={∆}_{u}/{∆}_{y}$$

where Δ_*y*_ is the displacement of the component at yield and the yield displacement (mm); Δ_*u*_ is the displacement of the component at the time of ultimate failure, and the ultimate displacement (mm).

### 3.5. Energy Dissipation

**Figure 13:**
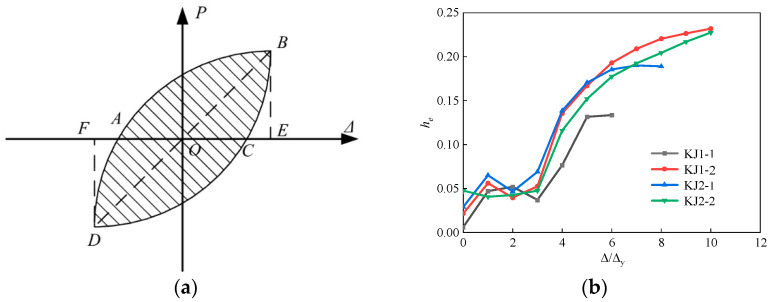
(**a**) Schematic illustration of calculating equivalent viscous damping coefficient, and (**b**) Results of equivalent viscous damping coefficient for each join specimen.

Equivalent viscous damping coefficient *h_e_* was used to analyze the energy dissipation of joints. Figure 13a schematically illustrates its calculation through Equation (8). The hysteresis curve of the specimen is decomposed into independent hysteresis loops. The larger the area surrounded by the hysteresis loop, the higher the equivalent viscous damping coefficient and the stronger the energy dissipation capacity of the structure or component.

(8) $$h_{e}=\frac{1}{2\pi }\frac{S_{ABCDA}}{S_{OBE}+S_{ODF}}$$

where *S_ABCDA_* is the energy consumed by the specimen during one full cycle of loading. *S_OBE_* and *S_ODF_* are the energy absorbed by the specimen during one full cycle of loading.

Figure 13b demonstrates the equivalent viscous damping coefficient of each beam-column joint with the loading level (i.e., Δ/Δ_*y*_). The equivalent viscous damping coefficient of each specimen showed a trend of decreasing first, followed by an increase. Such a decrease occurred when the stiffness of each specimen decreased, and the bearing capacity reached the cracking load threshold.

The decreasing interval of the equivalent viscous damping coefficient of the KJ1-1 specimen reached three loading levels. With the addition of steel fibers, the equivalent viscous damping coefficient of the KJ1-2 specimen decreased by one loading level and was followed by a rapid increase. The equivalent viscous damping coefficient finally reached 0.2320, which was 73.8% higher than that of the specimen KJ1-1. The results showed that the addition of steel fibers significantly improved the energy dissipation capacity of the beam-column joint. The working performance quickly recovered after the stiffness of the specimen decreased and the surface cracked. The equivalent viscous damping coefficient was significantly increased.

The equivalent viscous damping coefficient of specimen KJ2-2 showed a downward trend in the early loading stage, followed by a rapid increase after the 4th loading level. Its equivalent viscous damping coefficient finally reached 0.2257, which was 69.1% higher than that of specimen KJ1-1. In the case of no stirrups in the core area, the equivalent viscous damping coefficient of specimen KJ2-1 finally reached 0.1900, which was 42.3% higher than that of specimen KJ1-1. It indicates that the addition of steel fiber in the core area of the beam-column joint renders a similar or even better effect as the configuration of stirrups with respect to the energy dissipation capacity.

### 3.6. Stiffness and Strength Degradation

**Figure 14:**
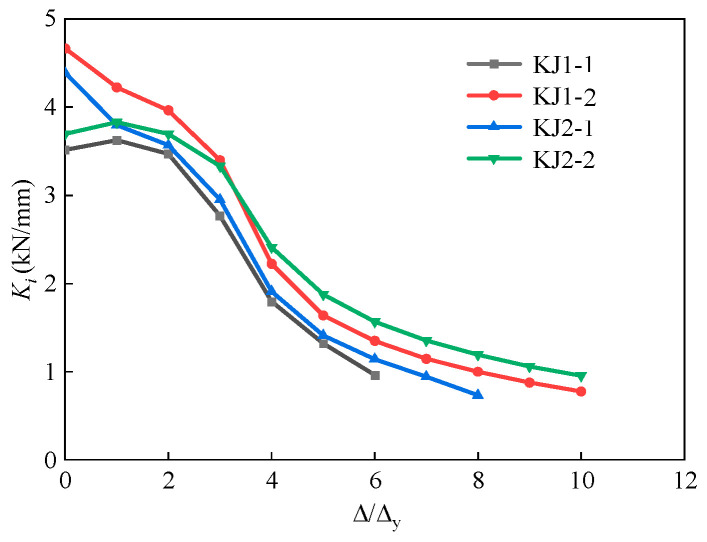
Results of secant stiffness of each specimen.

To better understand the stiffness degradation, the secant stiffness Ki was calculated through Equation (9) [32,33]. Figure 14 demonstrates the secant stiffness in each loading level.

(9) $$K_{i}=\frac{\sum_{j=1}^{n}P_{i,j}}{\sum_{j=1}^{n}{∆}_{i,j}}$$

where *P_i,j_* is the peak load under the *j*th cyclic loading with the *i* displacement amplitude; *Δ_i,j_* is the displacement value under the *j*th cyclic loading with the *i* load amplitude; *n* is the number of cycles.

Compared with KJ1-1, the initial stiffness of KJ1-2, KJ2-1, and KJ2-2 were increased by 32.7%, 24.8%, and 5.2%, respectively. The results showed that the initial stiffness of concrete beam-column joints was significantly improved by adding steel fiber into the joint core area. As the loading level increased, the stiffness degradation of each specimen accelerated gradually. In the late loading period, the slope of the stiffness degradation curve of the specimen with steel fibers was significantly lower than that of the specimen without steel fibers. This is due to the fact that, as the load increases, the specimen enters the yield stage, and cracks appear at the beam-end and core area of the joint. Steel fibers play the “bridging role” and delay the crack development rate of HSFRC specimens, lowering the stiffness degradation rate in the late loading period. However, without steel fiber in HSC, the whole specimen is relatively brittle, and the rate of stiffness degradation is higher than that of SFRHSC. Under the loading level of 6, the stiffness of specimens KJ1-2, KJ2-1, and KJ2-2 was 40.2%, 18.6%, and 62.8% higher than that of KJ1-1, respectively. An obvious improvement was presented.

**Figure 15:**
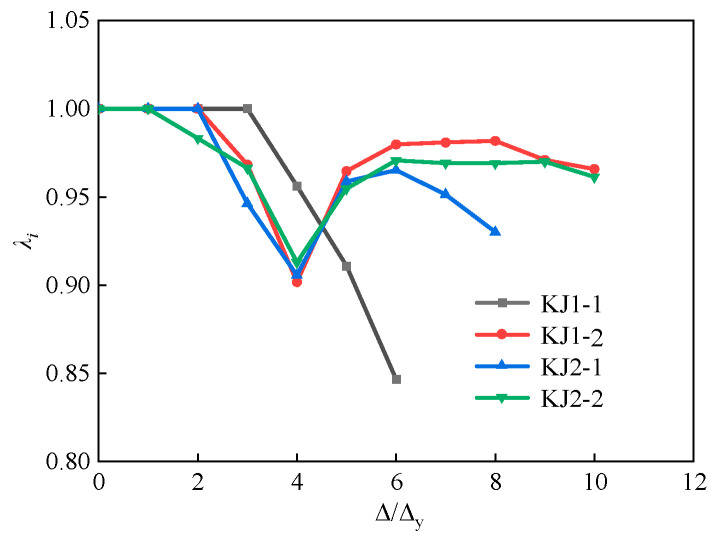
Comparison of strength degradation curves of each specimen.

The strength degradation ratio of each joint specimen was calculated through Equation (10). The calculated results are shown in Figure 15.

(10) $$\lambda_{i}=\frac{P_{i,j}}{P_{i,1}}$$

where *P_i,j_* is the peak load under the *j*th cyclic loading with the *i* displacement amplitude, and the average value of the peak load under positive and negative loading are taken; *P_i_*_,1_ is the peak load during the first cyclic loading under the *i* displacement amplitude, and the average value of the peak load under forward and reverse loading is taken.

The bearing capacity of KJ1-1 without steel fibers showed a decreasing trend with the increase in loading level. As for the other specimens containing steel fibers, the strength degradation ratio showed a trend of decreasing first, followed by an increase and a final decrease. The loading level at the point of trend transformation was the same as the cracking load interval of the specimens. For instance, the evolution of *λ_i_* for KJ1-2 changed from a decreasing trend to an increasing trend at the loading level of 4Δ_y_, which was the cracking stage of the specimen. It indicates that the working performance of the beam-column joints after cracking can be significantly improved by adding steel fibers into the core area of the joints. The steel fiber functions in the concrete beam-column joints and plays a “bridging role”, ensuring that the overall bearing capacity of the HSFRC joints continues to improve after cracking.

Additionally, the strength degradation ratio *λ_i_* of KJ2-1 and KJ2-2 finally reduced to 0.9300 and 0.9613, both of which were above 0.9. Compared to specimen KJ1-1, it can be found that steel fibers can effectively replace stirrups in the core area with respect to strength degradation. Partial replacement of stirrups exhibits superior performance compared to complete replacement. With respect to saving cost, adopting fibers in the joint core area instead of the entire beam-column joint significantly reduces the cost of construction. Partial replacement of stirrups with steel fibers can be more cost-effective than that of complete replacement due to the difference in the price of stirrups and steel fibers.

## 4. Simulations through Finite Element Modeling (FEM)

To further investigate the behavior of exterior HSFRC beam-column joint, pseudo-static loading tests were performed numerically through the finite element method (FEM). The concrete damaged plasticity (CDP) model for concrete beam-column joints under seismic loads has been employed by researchers to successfully analyze the structural performance of various concrete beam-column joints (including joints with and without fibers) [34,35]. As such, it is adopted in this simulation with the material properties obtained from the experimental results. The influence of the volume ratio of steel fibers and the stirrups ratio in the core area on the cyclic behavior of specimens was studied. In addition to these two parameters, the geometric dimensions (e.g., width and height) of the beam and column may affect the structural performance of the joint [36], which is not within the scope of the present study. The materials and dimensions adopted in the experimental and numerical investigations in this work are in accordance with the standards as cited in the corresponding context.

### 4.1. Constitutive of Materials

The double broken line constitutive model is used for the steel bars, with measured parameters of the mechanical properties (see Section 2.2). Two kinds of concrete, HSC, and HSFRC, are used in the numerical simulations in accordance with the experiments.

For HSC, the constitutive relation method given in standard GB50010-2010 [22] is employed. For HSFRC, the influence of steel fibers mainly lies in the peak stress and peak strain of concrete. In addition, the decreasing stage of the uniaxial compressive and tensile stress-strain curve of concrete is gentler. The constitutive curve of HSFRC is proposed in refs. [37,38] is used in this study. The mechanical parameters of HSFRC are obtained through experimental results, as shown in Section 2.2.

As an inhomogeneous material, concrete has anisotropy compared with other homogeneous materials. Under the action of earthquake load, concrete structures repeatedly bear compression and tension. The concrete damage plastic model (CDP) is employed in the simulation. It considers the difference in tensile and compressive properties of materials and can be applied to unidirectional loading. It can also be used for cyclic and dynamic loading tests. For the constitutive model of concrete plastic damage, the key is to determine the damage factor, which is derived based on ref. [39].

### 4.2. Frame Model and Boundary Conditions

**Figure 16:**
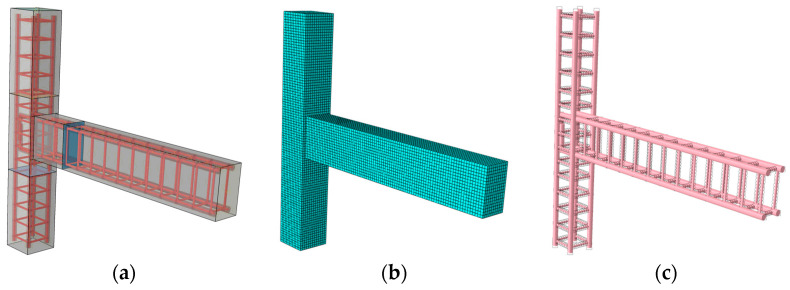
Diagram of (**a**) the beam-column joint model, (**b**) the concrete model, and (**c**) the reinforcement network.

The finite element simulation adopts the same loading system and boundary conditions of beam-column joints corresponding to experiments in Section 2.3. As shown in Figure 16, the steel bar skeleton is defined as being completely embedded in the concrete and forming contact with the concrete model to maintain a certain friction force for common force. Rigid pad blocks are placed on the top of the column, and the end of the beam of the specimen, and Tie constraints are adopted between the rigid pad block and the concrete. A reference point is set on each of the beam and column pad blocks to facilitate subsequent loading. Coupling constraints are adopted between the reference point and pad blocks.

The model’s loading protocol is divided into two steps. The first loading step is to apply a constant axial load (600 kN) to the top of the column. The second loading step is to apply cyclic load to the end of the beam. The displacement amplitude control is used to control the load on the beam-end, which can better simulate the stress form and damage change of the beam-column joint under the cyclic load. Under the load module, the model load and boundary conditions are defined. The boundary conditions for the top of the column are U1 = U2 = UR1 = UR3 = 0, the bottom of the column is U1 = U2 = U3 = UR1 = UR3 = 0, and the boundary conditions for the beam end are U1 = U2 = UR1 = UR2 = UR3 = 0.

As for the mesh division, the geometric model is arranged with seeds to control the cell density and position, and then the automatic algorithm is used to directly generate a mesh in the grid module to mesh the beam-to-column node model. After several trial calculations, the concrete model and the steel skeleton model adopt global distribution, and the mesh size is divided into 15 mm.

### 4.3. Verification

**Figure 17:**
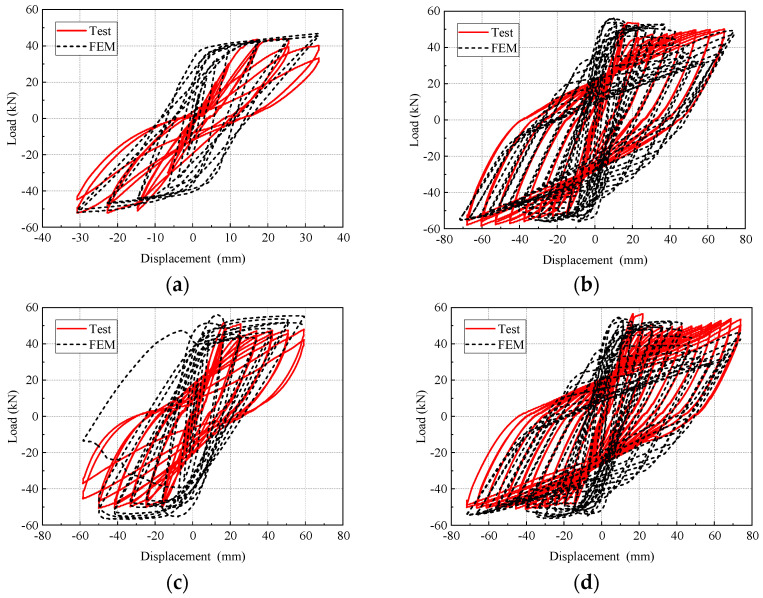
Comparison of numerical and experimental hysteresis curves for specimens (**a**) KJ1-1, (**b**) KJ1-2, (**c**) KJ2-1, and (**d**) KJ2-2.

Figure 17 presents the comparison between the numerical and experimental results of hysteresis curves for all four joint specimens. The simulated results demonstrate a good agreement with the experimental results. Table 7 compares the results obtained from numerical and experimental investigations. It can be observed that the ratio of the experimental-to-numerical strength (*P*_max-test_/*P*_max-number_) yields a mean of 1.01 and a *R*^2^ value of 0.99. The displacement ductility coefficient of experimental and numerical results demonstrates a *R*^2^ value of 0.93. The good agreement between the numerical and the experimental results in terms of hysteresis response, load, and displacement indicates the accuracy of the model in analyzing the seismic behavior for both HSC and HSFRC.

### 4.4. Parametric Study

#### 4.4.1. Effect of the Volume Ratio of Steel Fibers

SFRHSC beam-column joints with five different volume ratios of steel fibers (i.e., 0.0%, 0.5%, 1.0%, 1.5%, 2.0%) are investigated, which are denoted as FEA-0.0-0.6, FEA-0.5-0.6, FEA-1.0-0.6, FEA-1.5-0.6, FEA-2.0-0.6. All the geometric parameters are identical for all cases, and the stirrups ratio in the core area of the joint is 0.6%.

**Figure 18:**
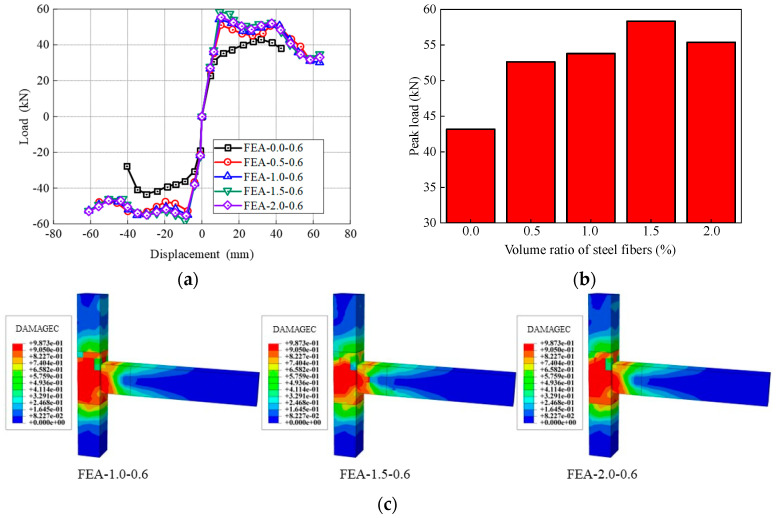
Influence of volume ratio of steel fibers on the (**a**) skeleton curves, (**b**) peak load, and (**c**) compression damage of concrete.

**Figure 19:**
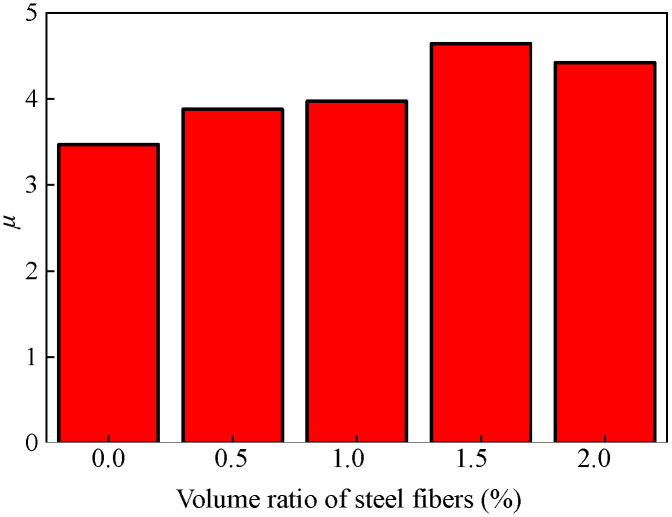
Influence of volume ratio of steel fibers on the displacement ductility coefficients.

Figure 18 shows the comparison of skeleton curves and peak load in the cases of different steel fiber volume ratios. There is little difference in the initial loading stage of the skeleton curves. As the loading level increases, the skeleton curves of FEA-0.0-0.6 decreases first. As the volume ratio increases from 0.0% to 1.5%, the peak load of the model increases correspondingly. The most significant increase in the peak load occurs when the volume ratio of steel fibers increases from 0.0% to 0.5%, which is 21.9%. Nevertheless, a decrease in the peak load is displayed with an increase in the volume ratio of steel fibers from 1.5% to 2.0%. Notedly, the peak load of specimen embedding 2.0% steel fibers is still 30.6% higher than the case of no steel fibers (FEA-0.0-0.6). This shows that the load-bearing capacity of the joint can be significantly improved by adding steel fibers in the joint core area. Nevertheless, there is no single relationship between the load-bearing capacity and the volume ratio of steel fibers. The highest value of peak load occurs in the case of a 1.5% volume ratio of steel fibers. Further increment of steel fibers in the core area decreases the peak load. This is due to the fact that a higher amount of steel fibers (e.g., volume ratio greater than 1.5%) decreases the compressive properties of concrete [37]. Studies have shown that a large amount of steel fibers induces poor mixing and consolidation of the concrete matrix, entrapping air in the concrete [40]. Observably, when the volume ratio of steel fibers is no more than 1.5%, the extent of concrete compression damage in the joint core area reduces with increasing the amount of steel fibers (see FEA-1.0-0.6 and FEA-1.5-0.6 in Figure 18c). However, as the volume ratio of steel fibers increases from 1.5% to 2.0%, the extent of concrete compression damage deteriorates. The displacement ductility coefficients present a similar trend, as shown in Figure 19.

**Figure 20:**
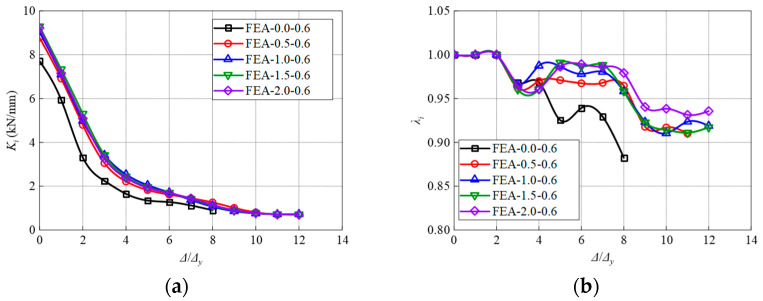
Influence of volume ratio of steel fibers on the (**a**) Stiffness and (**b**) Strength degradation.

Figure 20 depicts the results of secant stiffness (*K_i_*) and strength degradation ratio (*λ_i_*). The specimen FEA-0.0-0.6 demonstrates the lowest value and highest degradation rate of stiffness compared to other specimens. It can be concluded that increasing the volume content of steel fibers reduces the amplitude and rate of stiffness and strength degradation, maintains the overall stiffness stability of the model, and enhances the seismic performance.

#### 4.4.2. Effect of Stirrups Ratio in the Joint Core Area

SFRHSC beam-column joints with five different stirrups ratios in the joint core area (i.e., 0.0%, 0.3%, 0.6%, 0.9%, 1.2%) are investigated, which are denoted as FEA-1.5-0.0, FEA-1.5-0.3, FEA-1.5-0.6, FEA-1.5-0.9, FEA-1.5-1.2. All the other geometric parameters are identical for all cases, and the volume ratio of steel fibers in the core area is 1.5%.

**Figure 21:**
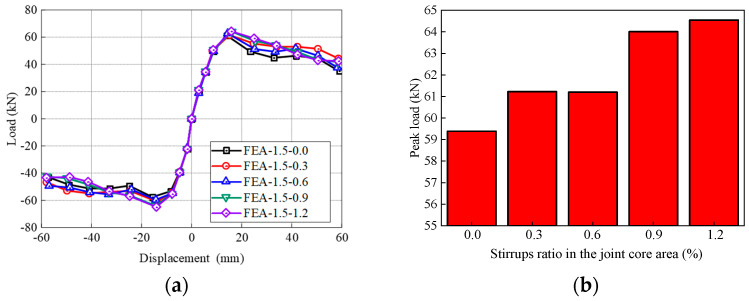
Influence of stirrups ratio on the (**a**) skeleton curves, (**b**) peak load.

Figure 21 shows the comparison of skeleton curves and peak load of different stirrups ratios in the joint core area. The skeleton curves of all models basically overlap in the elastic stage. The stirrups ratio mainly affects the strengthening stage and failure stage of the skeleton curves. Without stirrups in the core area, the skeleton curve of FEA-1.5-0.0 declines after reaching the peak load, rendering the highest degradation rate. With the increase of stirrups ratio in the core area, the peak load increases (Figure 21b). Compared with specimen FEA-1.5-0.0, the peak load with stirrups ratio of 0.3%, 0.6%, 0.9%, and 1.2% in the core area increases by 3.1%, 3.0%, 7.8% and 8.7%, respectively. Noticeably, when the stirrups ratio increases from 0.6% to 0.9%, the increment of the peak load is the largest at 4.6%. As the stirrup ratio increases from 0.9% to 1.2%, the peak load of FEA-1.5-1.2 is only 0.8% higher than that of FEA-1.5-0.9. This indicates that the load-displacement properties are in a positive relationship with the stirrups ratio in the joint core area. However, the increment amount is not increased with the increase in stirrup ratio. The optimal enhancement effect occurs when the stirrups ratio is 0.9% in the present study.

**Figure 22:**
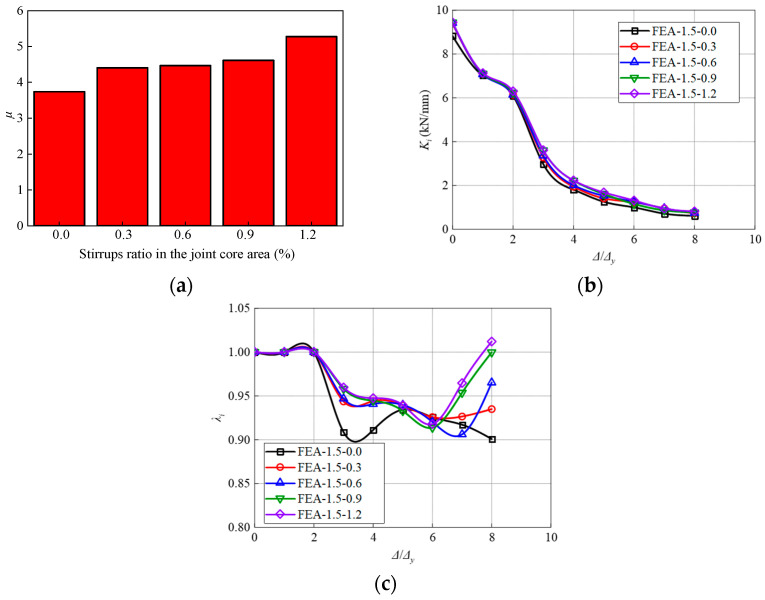
Influence of stirrups ratio on the (**a**) Displacement ductility coefficient, (**b**) Stiffness, and (**c**) Strength degradation.

A similar trend is observed with respect to the ductility, stiffness, and strength degradation (Figure 22). With the increase of the stirrup ratio, the ductility of the model is significantly improved (Figure 22a). This is due to the increase in the number of stirrups in the core area, which makes the overall stress of the steel skeleton more balanced, and the model bearing capacity is fully utilized. Additionally, increasing the proportion of stirrup in the core area significantly impedes the degradation of stiffness and strength. Nevertheless, it is found that when the stirrup ratio increases from 0% to 0.9%, the strength degradation coefficient increases by 11.0%, while the increment reduces to 1.2% in the case of the 1.2% stirrups ratio. The enhancement effect is not obvious after a 0.9% stirrups ratio.

## 5. Conclusions and Recommendations

The present study investigated the seismic performance of steel fiber reinforced high-strength concrete (SFRHSC) beam-column joint in comparison to high-strength concrete (HSC) joint. Four specimens, including three beam-column joints with steel fibers and one joint without steel fibers, were tested. Numerical simulations based on the finite element method were performed and validated by the experimental results. Parametric analyses were further conducted through numerical simulations. The following conclusions can be drawn from the results:

(1) Two failure modes were demonstrated, including joint core shear failure and beam-end flexural failure. The increase of stirrups and the addition of steel fibers in the joint core area change the failure mode of shear failure to flexural failure.
(2) The addition of steel fibers makes the hysteresis curve of the beam-column joints fuller and improves the load-bearing capacity, ductility, and energy dissipation capacity. Additionally, the seismic performance of joints in the cases of replacing part of or all the stirrups in the joint core area with steel fibers demonstrates similar results. Steel fibers not only enhance the seismic performance but also can replace the stirrups in the core area of the beam-column joints. Notedly, partial replacement of stirrups by steel fibers demonstrates superior seismic performance than complete replacement.
(3) Based on the parametric analyses through numerical simulations, it has shown that as the volume ratio of steel fibers in the core area increases from 0.0% to 1.5%, the peak load-bearing capacity increases by 35.2%, and the displacement ductility coefficient increases by 33.8%. In addition, the energy dissipation capacity gradually increases while the degradation rates of load-bearing capacity and stiffness significantly reduce. Nevertheless, as the volume ratio of steel fibers increases from 1.5% to 2.0%, the seismic performance decreases. The optimal volume ratio of steel fibers is concluded to be 1.5%.
(4) In the case of the SFRHSC joint containing 1.5% steel fibers, the seismic performance, including load-bearing capacity, ductility, and energy dissipation capacity, significantly improves as the stirrups ratio in the core area increases from 0% to 0.9%. As the stirrups ratio in the core area increases from 0.9% to 1.2%, the improvement in seismic performance is not significant (i.e., merely a 1.2% increment in strength degradation coefficient). Therefore, the optimal reinforcement ratio of the stirrups in the core area is proposed to be around 0.9% in this study.

In addition to the volume ratio of steel fibers and stirrups ratio, factors such as the strength of the concrete matrix and the type of steel fibers are also crucial to the seismic performance of beam-column joints. These factors can be analyzed using the finite element model proposed in this paper.

## Figures and Tables

**Table 1:** Details of beam-column joint specimens.

Specimens Notation	Axial Compression Ratio	Volume Ratio of Steel Fiber (%)	Length of Steel Fiber Mixed into Beam End (mm)	Number of Stirrups in the Joint Core Area	Stirrups Ratio in the Joint Core Area (%)
KJ1-1	0.2	0.0	250	5	0.6
KJ1-2	0.2	1.0	250	5	0.6
KJ2-1	0.2	1.0	250	0	0.0
KJ2-2	0.2	1.0	250	2	0.5

**Table 2:** Mixture proportion of concrete (kg/m^3^) and tested compressive strength.

Concrete Notation	Cement (P II 52.5)	Water	Coarse Aggregates	Fine Aggregates	Mineral Powder	Fly Ash	Admixture	Steel Fibers	Compressive Strength (MPa)
HSC	406	119	985	723	81	37	8.68	0	72.1
HSFRC	406	119	985	723	81	37	8.68	70 (1.0%)	84.2

**Table 3:** Mechanical properties of concrete.

Concrete Notation	Compressive Strength of Cubes (MPa)	Axial Compressive Strength (MPa)	Splitting Tensile Strength (MPa)	Modulus of Elasticity (MPa)
HSC	72.1	76.2	8.9	3.89 × 10^4^
HSFRC	84.2	80.9	11.5	3.80 × 10^4^

**Table 4:** Mechanical properties of steel reinforcements.

Reinforcement	Diameter (mm)	Yield Strength (MPa)	Ultimate Tensile Strength (MPa)	Elongation (%)	Modulus of Elasticity (MPa)
Stirrups	8	306.9	472.7	25	2.01 × 10^5^
Main rebar	20	422.7	585.1	30	2.09 × 10^5^

**Table 5:** Details of crack parameters.

Specimens Notation	Maximum Crack Width at the First Loading Level under Displacement Loading Control (mm)		Maximum Crack Width at Failure (mm)	
	Core Area	Beam-End	Core Area	Beam-End
KJ1-1	0.5	1.3	1.2	1.5
KJ1-2	0.1	1.0	0.2	Exposed steel
KJ2-1	Non-visible	1.0	8.0	15.0
KJ2-2	Non-visible	0.5	0.5	30.0

**Table 6:** Summary of experimental results.

Specimens Notation		*P*_cr_ (kN)	*P*_y_ (kN)	*P*_max_ (kN)	*P*_u_ (kN)	Δ*_y_* (mm)	Δ*_u_* (mm)	*μ*
KJ1-1	Forward	20.00	41.68	43.74	37.18	16.07	41.00	2.55
	Reverse	−20.00	−42.30	−52.20	−44.37	11.31	30.79	2.72
KJ1-2	Forward	30.00	52.65	53.70	50.17	19.31	68.76	3.56
	Reverse	−30.00	−52.01	−58.48	−57.91	18.93	67.88	3.59
KJ2-1	Forward	30.00	50.54	51.64	48.01	17.58	59.26	3.37
	Reverse	−30.00	−43.75	−50.59	−43.00	20.12	62.49	3.11
KJ2-2	Forward	30.00	53.59	56.55	53.45	22.36	65.48	3.31
	Reverse	−30.00	−46.83	−51.18	−50.19	21.81	64.57	3.29

Note: *P*_cr_ represents the test value of beam end load when the concrete in the core area of the beam-column joint cracks; *P*_y_ represents the yield load value of beam-column joints; *P*_max_ represents the peak load value of beam-column joints; *P*_u_ represents the test value of beam end load when the beam-column joint fails. The minus sign represents the reverse loading direction.

**Table 7:** Comparison of numerical and experimental results.

Specimens Notation		*P*_max-test_ (kN)	*μ*_test_	*P*_max-numer_ (kN)	*μ*_numer_	*P*_max_ _-test_ */P*_max_ _-numer_	*μ*_max_ _-test_ */μ*_max_ _-numer_
KJ1-1	Forward	43.74	2.55	44.04	2.17	0.99	1.18
	Reverse	−52.20	2.72	−50.32	2.64	1.04	1.03
KJ1-2	Forward	53.70	3.56	53.45	3.73	1.00	0.96
	Reverse	−58.48	3.59	−55.13	3.69	1.06	0.97
KJ2-1	Forward	51.64	3.37	50.37	3.53	1.02	0.95
	Reverse	−50.59	3.11	−53.21	3.37	0.95	0.92
KJ2-2	Forward	56.55	3.31	53.48	3.63	1.06	0.91
	Reverse	−51.18	3.29	−52.53	3.59	0.97	0.92
					Mean	1.01	0.98
					*R* ^2^	0.99	0.93

## Data Availability

The raw data supporting the conclusions of this article will be made available by the authors on request.
